# Cardiovascular effects of linalyl acetate in acute nicotine exposure

**DOI:** 10.1186/s12199-017-0651-6

**Published:** 2017-04-24

**Authors:** Ju Ri Kim, Purum Kang, Hui Su Lee, Ka Young Kim, Geun Hee Seol

**Affiliations:** 1 Department of Basic Nursing Science, School of Nursing, Korea University, Seoul, 02841 Republic of Korea; 2 Department of Nursing, School of Nursing, Gachon University, Incheon, 21936 Republic of Korea

**Keywords:** Linalyl acetate, Acute nicotine, Adolescent, Cardiovascular changes

## Abstract

**Backgroud:**

Smoking is a risk factor for cardiovascular diseases as well as pulmonary dysfunction. In particular, adolescent smoking has been reported to have a higher latent risk for cardiovascular disease. Despite the risk to and vulnerability of adolescents to smoking, the mechanisms underlying the effects of acute nicotine exposure on adolescents remain unknown. This study therefore evaluated the mechanism underlying the effects of linalyl acetate on cardiovascular changes in adolescent rats with acute nicotine exposure.

**Methods:**

Parameters analyzed included heart rate (HR), systolic blood pressure, lactate dehydrogenase (LDH) activity, vascular contractility, and nitric oxide levels.

**Results:**

Compared with nicotine alone, those treated with nicotine plus 10 mg/kg (*p* = 0.036) and 100 mg/kg (*p* = 0.023) linalyl acetate showed significant reductions in HR. Moreover, the addition of 1 mg/kg (*p* = 0.011), 10 mg/kg (*p* = 0.010), and 100 mg/kg (*p* = 0.011) linalyl acetate to nicotine resulted in significantly lower LDH activity. Nicotine also showed a slight relaxation effect, followed by a sustained recontraction phase, whereas nicotine plus linalyl acetate or nifedipine showed a constant relaxation effect on contraction of mouse aorta (*p* < 0.001). Furthermore, nicotine-induced increases in nitrite levels were decreased by treatment with linalyl acetate (*p* < 0.001).

**Conclusions:**

Taken together, our findings suggest that linalyl acetate treatment resulted in recovery of cell damage and cardiovascular changes caused by acute nicotine-induced cardiovascular disruption. Our evaluation of the influence of acute nicotine provides potential insights into the effects of environmental tobacco smoke and suggests linalyl acetate as an available mitigating agent.

## Background

Smoking is an independent risk factor for cardiovascular diseases, including atherosclerosis and ischemic heart diseases, by virtue of its negative effects on vascular endothelial function [33, 34] as well as pulmonary dysfunction [16, 20]. Most studies to date have focused on the mechanisms and pathophysiology of chronic diseases caused by smoking [3, 4, 12]. However, acute exposure, defined as a single exposure to a harmful substance [25], is an important issue in adolescents who begin to smoke voluntarily [11]. Adolescents are more vulnerable to neurobiological changes, mental health, and substance-use disorders, as this period of life is essential for brain development associated with self-control and regulation [9, 46]. Furthermore, adolescents who smoke have been reported to be at higher risk for cardiovascular disease than non-smoking individuals [15], and vulnerability to nicotine addiction has been reported higher in adolescents than in adults [14, 17, 35]. Despite the risks associated with adolescent smoking, the mechanisms underlying the effects of acute nicotine exposure on adolescents remain unknown. Animal models, especially adolescent rats aged 28–42 postnatal days, have been used to effectively investigate the pathophysiological effects of nicotine [40, 46].

Nicotine, one of the constituents of cigarettes, rapidly reaches the blood and brain after being absorbed through inhalation [2] and is thought to contribute to cardiovascular diseases caused by cigarette smoking [8, 28] and possibly the development of atherosclerosis [21]. The physiological effects of nicotine appear to differ depending on the dose, duration of exposure, and method of application [8]. A single exposure to nicotine has been reported to result in cognitive impairment, including impairments in learning and memory [14, 17, 35]. Acute exposure to nicotine has been reported to increase anxiogenic-like effects in rats and reduce behavioral pattern organizations, as shown by T-pattern analysis [5].

Linalyl acetate and linalool, the major constituent of several aroma essential oils, may regulate cardiovascular responses [13, 44]. A clinical trial reported that linalool decreases heart rate in healthy volunteers [27]. Linalyl acetate has been reported to evoke vascular smooth muscle relaxation in the rabbit carotid artery [26], and a recent study reported that bergamot essential oil, containing linalool and linalyl acetate as a main ingredient, induces blood vessel relaxation in the forced contracted mouse aorta [23]. In addition, linalyl acetate-rich lavender aromatherapy increases the coronary flow velocity reserve in healthy humans; thus, administration of lavender may improve coronary circulation [39].

Taken together, these observations provide support for the idea that linalool and linalyl acetate might alleviate acute nicotine-induced cardiovascular disabilities. Because of the difficulties associated with performing clinical studies, these effects were assessed in adolescent rats [46, 47]. There have been no systematic studies of the effects of linalool or linalyl acetate in nicotine-treated rats. Thus, the aim of the present study was to evaluate the mechanisms underlying the effects of linalyl acetate and linalool on cardiovascular changes in adolescent rats acutely exposed to nicotine.

## Methods

### Experimental protocol

Male Sprague-Dawley rats (5 weeks old) from Samtaco (Osan City, Korea) were used. The animals were housed in a temperature (22 °C ± 3 °C) controlled room with a 12-h light/dark cycle and were allowed free access to food and water. All experimental procedures were conducted in accordance with guidelines relevant to the care of experimental animals, as approved by the Institutional Animal Research Ethics Committee of Korea University (approval no. KUIACUC-2012-181), informed by the Guide for the Care and Use of Laboratory Animals published by the US National Institutes of Health (NIH Publication No. 85-23; revised 1996). All animals were acclimatized to laboratory conditions for 4 days and body weights were measured. Before experiments, heart rate and systolic blood pressure (sBP) were measured using a tail cuff and pulse transducer (AD Instruments, Sydney, Australia). Results are expressed as means of five data points. Each experimental group of rats was treated twice intraperitoneally, as follows: nicotine group (nicotine + vehicle); nicotine + linalool group; nicotine + linalyl acetate group; nicotine + nifedipine group. Control animals were intraperitoneally injected with vehicle (saline) instead of nicotine and component. After 30 min, heart rate and sBP were measured.

### Chemicals

(−)-Linalool, linalyl acetate, polyethylene glycol-200 (PEG-200), nifedipine, and nicotine were from Sigma (St. Louis, MO, USA). Linalool and was dissolved in PEG-200 and administered intraperitoneally (10 mg/kg) at a dose of 1.0 mL/kg. Linalyl acetate was dissolved in PEG-200 and administered intraperitoneally (1, 10, 100 mg/kg) at a dose of 1.0 mL/kg. Nifedipine was dissolved in dimethylsulfoxide and administered intraperitoneally (10 mg/kg) at a dose of 1.0 mL/kg. Nicotine was dissolved in distilled water and administered at a dose of 0.8 mg/kg [43]. Nicotine and essential oil components were injected intraperitoneally at separate sites.

### Measurement of lactate dehydrogenase (LDH) activity

Blood samples were collected after anesthetization with isoflurane and centrifuged at 3500 rpm for 15 min at 4 °C. Plasma LDH activity, a marker for cytotoxicity, was measured using a commercial LDH assay according to the manufacturer’s instructions (Roche Molecular Biochemicals, Mannheim, Germany).

### Preparation of mouse aortic rings and tension measurements

Male C57BL/6 mice (4–5 weeks, 17–21 g) were obtained from Samtaco (Osan City, Korea) and housed at a controlled temperature (24 ± 1 °C) with a 12 h light-12 h dark cycle and allowed free access to standard mouse chow and tap water. Mice were anesthetized by intraperitoneal injection of Zoletil (3:2 v/v) and Rompun (0.05 mg/g). The aortas were dissected, cleaned of surrounding connective tissue and fat in Krebs solution (118.3 mM NaCl, 4.78 mM KCl, 25 mM NaHCO_3_, 1.22 mM KH_2_PO_4_, 11.1 mM glucose, 2.5 mM CaCl_2_ and 2.5 mM MgCl_2_) and maintained at 37 °C in a 95% O_2_/5% CO_2_ atm. Each ring was placed in an isolated tissue bath (5 mL), with two strands of tungsten wire threaded through each ring; one strand was anchored in the organ bath chamber and the other connected to a mechanotransducer (Myo-Interface Model 620 M; DMT, Aarhus, Denmark). Before the experiments, an optimal resting tension (0.6–0.7 g) was applied for 30 min to ensure stabilization. The dose of each agent chosen in this experiment was based on previous findings [22, 26, 32].

### Nitrate assay

The accumulation in blood of nitrites, which are products of nitric oxide (NO), was measured by nitrite assays. Nitrite was determined after adding 100 μl Greiss reagent (0.1% naphthylethylenediamide and 1% sulfanilamide) to a 50 μl plasma sample. Nitrite concentrations were determined spectrophotometrically at 540 using a microplate reader.

### Statistical analysis

All statistical analyses were performed using SPSS software (version 23.0). The data were expressed as means ± S.E.M. and compared using one-way analysis of variance (ANOVA), followed by the Tukey LSD post hoc test. Significance was defined as a *p* < 0.05.

## Results

### Effects of linalyl acetate and linalool on HR and sBP in rats acutely exposed to nicotine

**Fig. 1:**
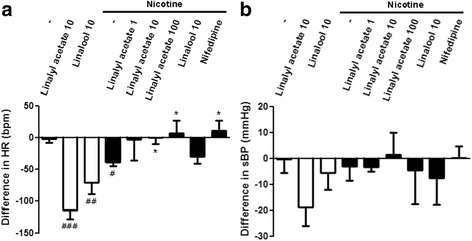
Linalyl acetate- and linalool-induced cardiovascular effects in nicotine-untreated and treated rats. **a** HR and **b** sBP are expressed as the difference between pre-test and post-test. Data represent mean values ± S.E.M. (*n* = 5–13 per group). ^###^ *p* < 0.001, ^##^ *p* < 0.01, ^#^ *p* < 0.05 compared with the control group; * *p* < 0.05 compared with nicotine group. HR: heart rate; sBP: systolic blood pressure

Although exposure of rats to a single dose of nicotine (co-administered with vehicle) significantly reduced HR (−39.16 ± 5.68 bpm, *p* = 0.044), pretreatment with 10 mg/kg (−0.53 ± 10.14 bpm, *p* = 0.036) and 100 mg/kg (6.62 ± 20.28 bpm, *p* = 0.023) linalyl acetate and nifedipine (10.34 ± 15.95 bpm, *p* = 0.021) resulted in a significant recovery of HR compared with nicotine alone (Fig. 1a). In contrast, acute treatment with nicotine did not significantly alter sBP (expressed as mmHg); pretreatment with linalyl acetate, linalool or nifedipine also had no effect (Fig. 1b).

### Effect of linalyl acetate on LDH activity in acute nicotine-treated rats

**Fig. 2:**
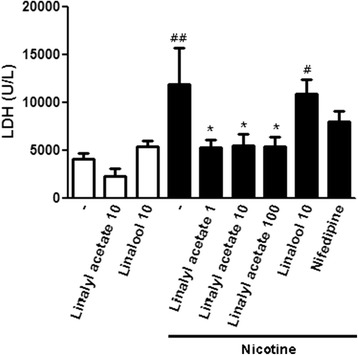
Effect of linalyl acetate and linalool on lactate dehydrogenase (LDH) activity in nicotine-untreated and treated rats. LDH on serum was measured to evaluate cell damage. Data represent mean values ± S.E.M. (*n* = 4–6 per group). ^##^ *p* < 0.01, ^#^ *p* < 0.05 compared with the control group; * *p* < 0.05 compared with nicotine group

Serum LDH activity, a marker of cell damage, was significantly higher in acute nicotine-treated than in control rats (11869.69 ± 3806.77 U/L vs. 4045.00 ± 659.61 U/L, *p* = 0.002) (Fig. 2). Compared with nicotine-treated rats, those treated with nicotine plus 1 mg/kg (5273.79 ± 825.82 U/L, *p* = 0.011), 10 mg/kg (5483.18 ± 1201.84 U/L, *p* = 0.010), and 100 mg/kg (5319.21 ± 1011.87 U/L, *p* = 0.011) linalyl acetate had significantly lower LDH activity. In contrast, LDH activity was similar in rats treated with nicotine alone and nicotine plus 10 mg/kg linalool (10910.3 ± 1484.50 U/L, *p* = 0.699). LDH activity was similar in control rats and rats treated with linalyl acetate or linalool.

### Vascular-related effects of linalyl acetate on nicotine exposed experimental rats

**Fig. 3:**
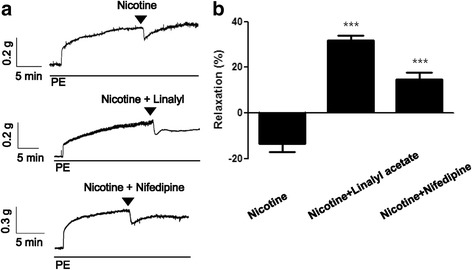
Vascular-related effects of linalyl acetate plus nicotine. **a** Original trace of tension measurements. **b** Relaxation effects of nicotine, with or without linalyl acetate, on phenylephrine (PE)-induced contractions in mice. *** *p* < 0.001 compared with nicotine group. PE: phenylephrine

The effects of nicotine plus linalyl acetate on the vascular system were evaluated in mouse aortic rings induced to contract by treatment with 10 μM phenylephrine (PE). Treatment of these aortic rings with 1 mM nicotine induced a slight relaxation effect, followed by a sustained recontraction. In contrast, treatment with 1 mM nicotine plus 300 μM linalyl acetate or 1 μM nifedipine showed a constant relaxation effect (Fig. 3). The relaxation rates with nicotine, nicotine plus linalyl acetate and nicotine plus nifedipine were −13.52 ± 3.62, 31.80 ± 2.22, and 14.70 ± 2.94, respectively, *p* < 0.001.

### Effect of linalyl acetate on nitric oxide (NO) in acute nicotine-treated rats

**Fig. 4:**
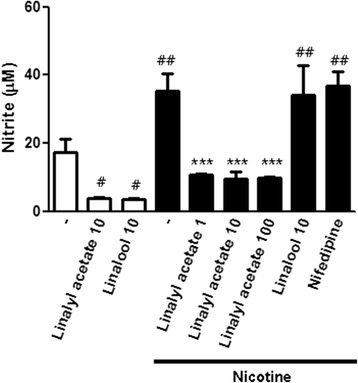
Effect of linalyl acetate and linalool on serum nitrite concentration in nicotine-untreated and treated rats. The concentrations of nitrite were measured using nitrite assay. Data represent mean values ± S.E.M. (*n* = 5–6 per group). ^##^ *p* < 0.01, ^#^ *p* < 0.05 compared with the control group; *** *p* < 0.001 compared with nicotine group

Next, we evaluated the effect of linalool and linalyl acetate on NO production in nicotine-treated rats by measuring nitrite, a product of NO and an indicator of NO levels. As shown in Fig. 4, nitrite levels were decreased in the nicotine + 1, 10 and 100 mg/kg linalyl acetate group (1 mg/kg, 10.62 ± 0.52 μM, *p* < 0.001; 10 mg/kg, 9.60 ± 1.98 μM, *p* < 0.001; 100 mg/kg, 9.87 ± 0.09 μM, *p* < 0.001) compared with those in the nicotine group (35.29 ± 5.02 μM).

## Discussion

In the present study, we investigated the effect of linalyl acetate and linalool on cardiovascular changes caused by nicotine. Acute nicotine exposure in these rats caused cell damage and vascular change, having no significant clinical implications including in regard to blood pressure and heart rate. Treatment of these rats with linalyl acetate, however, resulted in significant recovery of HR and LDH activity. There are conflicting reports on the vascular changes of acute nicotine. In a previous study, environmental smoke exposure was shown to induce an increase in central wave reflection and enhance microvascular relaxation. However, the relaxation effect was attenuated with repeated smoke exposure [1]. The cardiovascular reaction to nicotine may depend on the duration of exposure to nicotine. In our study, acute treatment with nicotine significantly reduced heart rate, an effect that was reversed by linalyl acetate, but not by linalool, suggesting that linalyl acetate affects the autonomic nervous response. This result is consistent with a previous study showing that foot-bath treatment with lavender, a linalyl acetate-rich essential oil, delayed changes in the balance of autonomic activity in healthy individuals [36]. Thus, it is possible that linalyl acetate contributes to autonomic balance. Environmental tobacco smoke is a contributor to cardiovascular disease as potent as active smoking. Passive smoking by the nonsmoker impairs vascular endothelial function and is associated with oxidative stress [18, 24]. Cigarette smoking disrupts the normal functioning of the autonomic nervous system, regardless of duration and direct-versus-indirect exposure [10]. Moreover, short-term second-hand smoking affects the cardiovascular autonomic nervous system and decreases heart rate variability [7]. In fact, second-hand smoke is considered a critical risk factor in cardiovascular disease. As noted above, it is likely that linalyl and linalyl-rich essential oils contribute to autonomic nervous system balancing. Thus, linalyl acetate or linalyl acetate-rich essential oils may be considered alternative agents for reducing nicotine-induced cardiovascular risk. However, in contrast to linalyl acetate, linalool did not induce cardiovascular changes in acute nicotine treated rats, suggesting that linalyl acetate may have specific cardiovascular effects during acute nicotine exposure. Moreover, although the reference concentration of linalyl acetate was effective, the same concentration of linalool was cytotoxic in this study.

Despite the importance of acute nicotine exposure of adolescents, the underlying mechanisms remain unknown due to the lack of clinical changes and the difficulty of performing studies in healthy individuals. The present study found that acute nicotine treatment of 5-week old rats resulted in significant changes in LDH, which is released during tissue damage, and vascular contractility. Moreover, using contracted mouse aortic rings, we found that acute nicotine showed a slight relaxation effect, followed by a sustained recontraction phase, whereas nicotine plus linalyl acetate showed a constant relaxation effect. This result is consistent with the observation showing that linalyl acetate induced vascular relaxation in rabbit blood vessels [26]. Similar effects were observed using the Ca^2+^ channel blocker, nifedipine. Since nicotine induces Ca^2+^ influx [30, 45], the relaxation effect of linalyl acetate is related to its inhibition of Ca^2+^ channels. Moreover, blood pressure is controlled by heart rate and systemic vascular resistance [6]. Thus, by simultaneously regulating peripheral resistance and heart rate, linalyl acetate and nicotine had no net effect on blood pressure. Nitric oxide is an intracellular mediator and has principle role in several physiological responses. And it is produced by nitric oxide synthesis (NOS) such as, constitutive NOS (located in the vascular endothelium, eNOS) and inducible NOS (iNOS). Particularly, iNOS synthesis NO involved pathophysiological processes of several cardiovascular disorder [31, 38]. Nicotine has been suggested that it markedly enhanced the iNOS in the context of inflammation [42]. Moreover, nicotine is known to contribute to endothelial dysfunction [41]. One study reported that endothelial dysfunction causes the imbalance between iNOS and eNOS in acute ischemic renal injury [19]. In the current study, linalyl acetate alleviated the observed effect of acute nicotine on NO, reducing NO to the same level as that in the control group. Therefore, linalyl acetate might act to interrupt the NOS enhancing effects of nicotine.

Adolescents are in a critical stage of brain development associated with neuronal signaling and cognitive function and are vulnerable to acute nicotine exposure [46]. This exposure may be better prevented and treated by palliative and supportive methods, including aromatherapy, than by aggressive medical management. Essential oils that contain linalyl acetate and linalool, such as *Citrus bergamia* (bergamot), *Lavandula angustifolia* (lavender) and *Salvia sclarea* (clary sage), have been reported to influence the cardiovascular system. In a clinical study, inhalation of clary sage essential oil, which contains considerable linalyl acetate, was reported to decrease blood pressure in female patients [37]. *Lavandula angustifolia* is known to decrease tone in rat skeletal muscle and relax rabbit smooth muscle [26, 29]. Thus, these essential oils may be considered potential abirritants. However, the molecular mechanism underlying the cardiovascular effects of linalyl acetate remains poorly understood, suggesting a need for additional studies of markers of cardiovascular risk.

## Conclusions

Taken together, linalyl acetate reversed cell damage and cardiovascular changes, including heart rate and production of NO components, of acute nicotine-induced cardiovascular disruption. Our evaluation of the influence of acute nicotine provides potential insights into the effects of environmental tobacco smoke and suggests linalyl acetate as an available mitigating agent.

## Abbreviations

HR: Heart rate
LDH: Lactate dehydrogenase
NO: Nitric oxide
NOS: Nitric oxide synthesis
PE: Phenylephrine
Sbp: Systolic blood pressure
